# Identifying and treating maternal mental health difficulties in Afghanistan: A feasibility study

**DOI:** 10.1186/s13033-020-00407-1

**Published:** 2020-10-27

**Authors:** Mark Tomlinson, Deepika Chaudhery, Habibullah Ahmadzai, Sofía Rodríguez Gómez, Cécile Rodríguez Gómez, Thandi van Heyningen, Mickey Chopra

**Affiliations:** 1 Department of Global Health, Institute for Life Course Health Research, Stellenbosch University, Cape Town, South Africa; 2 School of Nursing and Midwifery, Queens University, Belfast, UK; 3 World Bank, Health, Nutrition and Population Global Practice, South Asia Region, Kabul, Afghanistan; 4 Mental Health and Care Practices, Gender and Protection Department, Action Against Hunger, Kabul, Afghanistan; 5 Division of Epidemiology and Biostatistics, School of Public Health and Family Medicine, University of Cape Town, Cape Town, South Africa; 6 World Bank, Washington, USA

## Abstract

**Background:**

The disproportionately high burden of mental disorders in low- and middle-income countries, coupled with the overwhelming lack of resources, requires an innovative approach to intervention and response. This study evaluated the feasibility of delivering a maternal mental health service in a severely-resource constrained setting as part of routine service delivery.

**Methods:**

This exploratory feasibility study was undertaken at two health facilities in Afghanistan that did not have specialist mental health workers. Women who had given birth in the past 12 months were screened for depressive symptoms with the PHQ9 and invited to participate in a psychological intervention which was offered through an infant feeding scheme.

**Results:**

Of the 215 women screened, 131 (60.9%) met the PHQ9 criteria for referral to the intervention. The screening prevalence of postnatal depression was 61%, using a PHQ9 cut-off score of 12. Additionally, 29% of women registered as suicidal on the PHQ9. Several demographic and psychosocial variables were associated with depressive symptoms in this sample, including nutritional status of the infant, anxiety symptoms, vegetative and mood symptoms, marital difficulties, intimate partner violence, social isolation, acute stress and experience of trauma. Of the 47 (65%) women who attended all six sessions of the intervention, all had significantly decreased PHQ9 scores post-intervention.

**Conclusion:**

In poorly resourced environments, where the prevalence of postnatal depression is high, a shift in response from specialist-based to primary health care-level intervention may be a viable way to provide maternal mental health care. It is recommended that such programmes also consider home-visiting components and be integrated into existing infant and child health programmes. Manualised, evidence-based psychological interventions, delivered by non-specialist health workers, can improve outcomes where resources are scarce.

## Background

Approximately 4.4% of the world’s population suffer from depression and prevalence is increasing [1]. The highest proportion (more than 70%) of mental disorder lies in low- and middle-income countries (LMICs), where the treatment gap (the difference between the number of people who need care and those who receive care) for mental disorders is estimated to be between 76 and 85% [2]. Globally, 78% of deaths by suicide occur in LMICs [1]. In LMICs that have experienced conflict in the past two decades, a recent systematic review and meta-analysis found that the prevalence of mental disorders was 22%, substantially elevated above global prevalence estimates [3].

Key determinants of mental disorders include poverty, income inequality, interpersonal and collective violence, and forced migration [4]. Social determinants include demographic, environmental, sociocultural and socioeconomic factors. Women living in poverty, extreme adversity and facing multiple threats are most vulnerable [5, 6]. During the perinatal period, mental disorders may have significant deleterious effects on women and their children. Importantly, the prevalence rate of depression during this period for mothers in LMICs is substantially higher than the 10–13%, reported in HICs [7]. High rates of untreated maternal mental disorders coupled with adverse socioeconomic conditions contribute to poor maternal health, reduced maternal functioning, and compromised child health and development outcomes [8]. Maternal depression is associated with maternal morbidity, mortality and disability [9], and there are well-documented, negative outcomes for the psychosocial, cognitive, and socioeconomic development of offspring [10]. The low detection and treatment rate of perinatal mental disorders in LMICs may be mitigated by the integration of routine screening into primary care maternity and infant services.

A major factor affecting mental health service provision and uptake in LMICs is the overwhelming lack of resources. An initial step in the provision of services is mental health screening. Screening may be employed as a strategy for improving detection, for treatment referral or for allocation of resources in settings where mental health services are scarce [11]. Appropriate screening instruments can be used for either initial screening or for case-finding and may then be followed up with a more in-depth, time-intensive diagnostic enquiry [8]. For routine screening, the outcomes of over-diagnosis or under-diagnosis (as determined by sensitivity and specificity) require careful consideration. High sensitivity may result in over-diagnosis, as large numbers of people will be identified as requiring treatment, and potentially overwhelm an already overburdened health system. Also, where tools have not been culturally adapted this may lead to over or under diagnosis [9]. In addition, where limited services are available false-positive referrals will result in unnecessary exertion of resources [8].

There is robust evidence that perinatal depression and other common mental disorders can be effectively managed with psychological treatments. In LMICs, where access to mental health specialists is limited, evidence-based interventions delivered by non-mental health specialists, can improve outcomes for women with postnatal depression (PND) [12]. There are various manualised, short-term psychological interventions that have been developed in LMICs, which have a good evidence base in treating common perinatal mental disorders (CPMD). Based on Cognitive Behavioural Therapy, the Thinking Healthy Programme (THP) is one such intervention that has demonstrated successful treatment of CPMD [13, 14].

## Maternal mental health in Afghanistan

Afghanistan is one of the poorest countries globally, ranking second to last in the UN’s Human Development Index [10]. Nearly half (42%) of the population live below the poverty line [15] and there are high levels of food insecurity, hunger and undernutrition [16]. Despite an increase in the number of health facilities, midwives, community health workers (CHWs) and services available for pregnant women, new mothers and their infants [17], the percentage of unattended births remains high [18]. In 2015, only half of all births in the country were attended by a health professional [19].

In the light of these high levels of poverty, lack of autonomy, high rates of violence, and assaults on physical health, mental health problems are nearly twice as common amongst women [20]. Mental and behavioural disorders are the largest contributors to the number of years lived with disability (YLD), accounting for 22.7% of all YLD [21]. Depression is the most common of the mental health challenges [22] with the highest YLD (3.7%) and is about twice as common among women, compared to men [23]. Mental health legislation was introduced as early as 1987, but only recently has treatment coverage become a practical priority [24]. In general, mental health data for the country remain scant [24], and although a handful of studies have consistently found high rates of depression and anxiety amongst women [25, 26], mental health services are lacking. Furthermore, women’s ability to access health care is compromised by factors such as upholding family’s honour leading women to conceal mental health problems and failing to seek care. Further, social support networks for women may be limited, given the extreme limitations on their mobility and autonomy.

The aim of this study was to evaluate the feasibility of delivering a maternal mental health service in a severely-resource constrained setting as a part of routine service delivery with non-specialist health workers. This included training primary-level health workers to screen postpartum women for depression and to deliver appropriate treatment to those who are screened positive.

## Methods

### Design

The study was an exploratory feasibility study.

### Setting

The study was undertaken at two health facilities in the Parwan Province of Afghanistan: the Gul-Bahar Basic Health Center and the Sar-e-House Comprehensive Health Clinic.

### Study population

The study targeted women who had delivered a child within the last 12 months. Inclusion criteria were age (participants had to be over 18 years of age), and ability and willingness to provide informed consent. Participants also had to be fluent in Dari and to have the time and ability to complete the full interviews and participate in the intervention.

### Sampling strategy

All women who presented at either health facility and who had given birth within the past 12 months were invited to participate to be screened. Women who scored above the cut-off were invited to participate in the Thinking Healthy intervention.

### Measures

The PHQ-9 was used to screen women for depressive symptoms. The PHQ-9 is a nine-item depression screening tool, developed from the Primary Care Evaluation of Mental Disorders Procedure (PRIME-MD) for use in primary care settings [27, 28]. It has been tested for validity among diverse populations [29] including antenatal and postnatal women [27]. Scores are interpreted as follows: 0–4 normal, 5–9 mild depressive symptoms, 10–14 moderate depressive symptoms, > 15 moderately severe to severe depressive symptoms. Demographic, socio economic and psychosocial data was collected through self-reporting measures.

### Intervention

The psychological intervention used was an adaptation of the World Health Organisation’s manual “Thinking Healthy: A manual for psychosocial management of perinatal depression”, based on Atif Rahman’s Thinking Healthy Programme. The Thinking Healthy Programme is an evidence-based intervention for perinatal depression which has been shown to reduce symptoms of depression and increase women’s functioning [13]. It follows a cognitive behavioural approach and can be adapted to suit various contexts, depending on available resources. The structure and number of sessions were adapted for the Afghan context through input from various stakeholders including doctors and nurses in the primary health care system, community members, perinatal women, representatives from the World Bank, technical experts on intervention for maternal depression from Action Against Hunger, and an external expert on maternal depression.

### Study procedure

The study was implemented by Action Against Hunger (AAH). AAH is a global humanitarian and development organization that focuses on hunger and emergencies with more than 20 years of experience in Mental Health and Care Practices, including Support to Pregnant and Lactating Women and working on perinatal mental health.

### Training

AAH’s master trainers trained health facility staff and Provincial Public Health Directorate staff on the Integrated Management of Acute Malnutrition (IMAM) and Infant and Young Child Feeding (IYCF), as well as the Thinking Healthy post-partum depression protocol. A six-day training on IMAM and IYCF was conducted in March and April 2019. In April 2019 a four-day training on the Thinking Healthy Intervention was conducted. A total of 10 members of staff from the Ministry of Public Health (MoPH), and two AAH supervisors were trained to screen patients, collect data and administer the intervention.

### Data collection

There were ten members of staff, and two AAH supervisors who were trained to screen patients, collect data and administer the intervention. Post-intervention screening was administered following the end of the THP in order to evaluate impact. In addition to quantitative data collection, a qualitative, process evaluation was conducted in order to assess the feasibility of both the screening and the Thinking Healthy intervention.

### Ethics

A standard script was used to explain the study to participants as well as assurance about confidentiality and freedom to refuse participation or withdraw from the study at any time. The study included a comprehensive Standard Operating Procedure for suicidality. Women identified at risk were referred to the doctor from the same health centre (primary care level) which referred the patients to Parwan Provincial Hospital (which had doctors and psychologists). If women refused to go the hospital (mainly because of the distance), the doctor for the local health center did the follow-up. Ethics approval for the study was granted by the Institutional Review Board of the Ministry of Public Health, Afghanistan on March 17, 2019.

## Results

Between 14 April and 3 July 2019, 215 women were screened and 131 scored above the study’s PHQ-9 cut-off of 12. All 131 women agreed to participate in the Thinking Healthy intervention, but only 72 (55%) actually participated, with 47 (65%) completing all six sessions. The average age of women was 26 years, and the majority were married. Two-thirds (66%) of all women were exclusively breastfeeding and 9% of the infants met the criteria for moderate or severe acute malnutrition.

### Prevalence of depressive symptoms on the PHQ9

**Fig. 1:**
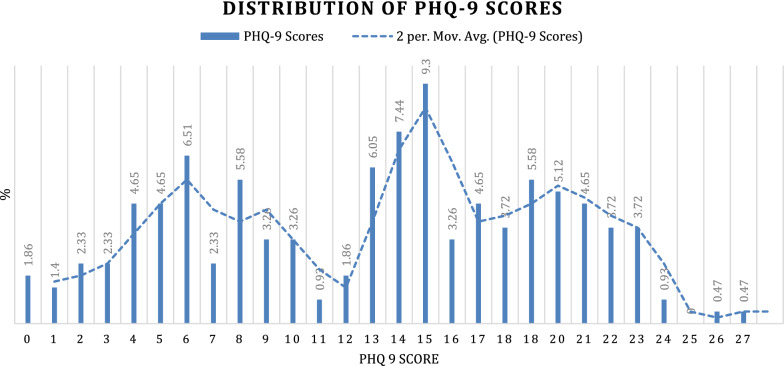
Distribution of PHQ-9 scores

Almost 87% of 215 women presented with some symptoms of post-partum depression (PPD) (ranging from mild to severe) using the PHQ9. See Fig. 1 below depicting the distribution of PHQ-9 scores. The percentage of women with mild depression (PHQ9 score 4–9) was 22%; moderate depression (PHQ9 score 10–14) was 20%; moderate-severe depression (PHQ9 score 15–19) was 27% and severe depression (PHQ9 score > 20) was 19%.

At the recommended PHQ9 cut-point of 10 and above, 65% of women screened positive. Using this study’s cut-point of 12 and above, 61% of participants screened positive for depression. With a cut-point of 15 or above, which was also considered for this study, 46% screened positive. In this study, we decided to use a cut-point of 12 (given that the average PHQ-9 score amongst the participants was 12.9, and it also fitted within the “moderately depressed” category. The PHQ-9 standardized severity grid was used as reference. Additionally, 29% of women screened indicated positive for suicidal ideation through their answers to question 9 of the PHQ-9.

A score of $\ge$ 5 indicates symptoms of depression ranging from 5–9 (mild), 10–14 (moderate) 15–19 (moderately severe) and > 20 (severe).

### Demographic and psychosocial variables associated with depressive symptoms

Mothers of children with moderate or severe acute malnutrition were more likely to have depressive symptoms. Psychosocial factors that were significantly associated with PHQ9 scores greater than 10 were feelings of anxiety as well as vegetative and mood symptoms including fatigue, variation in appetite and sleeping, sadness and irritability. Marital dispute, intimate partner violence, isolation, trauma and acute stress were also associated with depressive symptoms, as was grief associated with experience of loss (Table 1).

**Table 1:** Demographic and psychosocial variables associated with symptoms of depression

	Total sample N = 215 n (%)	Not depressed PHQ9 < 12 N = 84 (39%)	Depressed PHQ9 $\ge 12$ N = 131 (61%)	*p*-value
Age (average in years, SD)	26.3 (5.4)	25.8 (5.1)	27.1 (5.7)	0.055
Exclusively breastfeeding	86 (65)	21 (64)	65 (66)	0.833
Child malnutrition	12 (6)	2 (2)	10 (10)	0.014
Anxiety	84 (39)	18 (15)	66 (67)	< 0.001
Trauma	28 (13)	4 (3)	24 (24)	< 0.001
Sleep disturbance	98 (46)	17 (15)	81 (83)	< 0.001
Fatigue	116 (54)	28 (24)	88 (90)	< 0.001
Variation in appetite	104 (48)	19 (16)	85 (87)	< 0.001
Irritability	77 (36)	14 (12)	63 (64)	< 0.001
Headaches	65 (30)	13 (11)	52 (53)	< 0.001
Subjective feeling of sadness	93 (43)	18 (15)	75 (77)	< 0.001
Marital dispute	40 (19)	7 (6)	33 (34)	< 0.001
Emotional distress	75 (35)	15 (13)	60 (61)	< 0.001
Self-reported postnatal depression	124 (58)	31 (27)	93 (95)	< 0.001
Grief/Loss	74 (34)	18 (15)	56 (57)	< 0.001
Intimate partner violence	30 (14)	6 (5)	24 (24)	< 0.001
Emotional distress of child	67 (31)	14 (12)	53 (54)	< 0.001
Psychosomatic complaints	60 (28)	12 (10)	48 (19)	< 0.001
Social isolation	64 (30)	13 (11)	51 (52)	< 0.001
Acute stress	28 (13)	4 (3)	24 (24)	< 0.001

### Attendance at thinking healthy sessions

Of the 131 women who scored above the PHQ-9 cut-off of 12 which was used in the study, all signed the consent letter to participate in the Thinking Healthy sessions. However, only 72 (55%) returned to participate in the first session, and this number reduced each week by between 14 and 3% until the final session, when there were 47 (31%) women in attendance. Key reasons cited for missing sessions included child illness (n = 8), religious or cultural events (n = 4), prohibition by family (n = 3) and other reasons (n = 5) respondents. Of those who responded ‘other reasons’, one was not satisfied by the quality of services, one was referred to another hospital for treatment and one claimed that staff were not present at the HF. The two remaining respondents were unable to attend due to personal issues.

### Post intervention screening

**Fig. 2:**
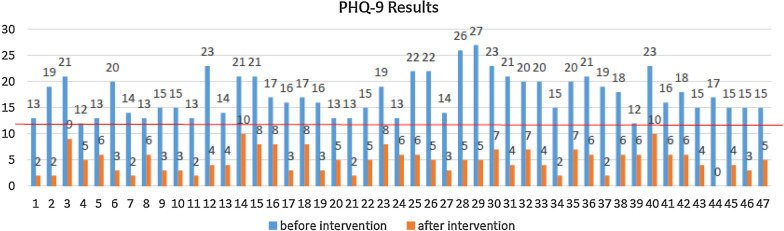
Comparison of PHQ-9 results before and after the THP intervention (n = 47)

All 47 women who completed all the Thinking Health Sessions were again screened with the PHQ-9 (see Fig. 2). All scores decreased by at least 6 points, with an average decrease of 13 points, and all scored below the study’s cut-off score of 12.

## Discussion

In this feasibility pilot study conducted in two health centres in Afghanistan, 87% of post-partum women screened with the PHQ9 presented with symptoms of depression. From these data, it is apparent that the overwhelming majority of women experience mental distress, indicative of chronic, sub-optimal maternal mental health. Such a high prevalence presents a significant public health concern and has important implications for maternal and child health interventions. Previously published research confirms that maternal mental illness predicts unfavourable outcomes for children’s physical and psychological development [30, 31]. Targeting maternal mental health has previously been identified as an effective way to mitigate the unfavourable impact of social adversity on child health and development [32]. Without simultaneously addressing maternal mental health, the outcomes of interventions designed to improve child nutrition may be jeopardised. Despite the scant literature published from post-conflict zones, there is enough evidence to confirm that women face an increased risk for CPMD and that maternal mental health should be prioritized and integrated into maternal and child health policy and practice. In order to address the wide gap between those requiring and those receiving treatment, the intervention approach needs to shift from facility-based screening and treatment to a community-based, universally applied public health campaign, that includes prevention and promotion components.

In many approaches, screening forms the first part of a stepped-care intervention [33]. This works well in areas that have sufficient resources and an established referral protocol. However, where there is extremely high prevalence coupled with poor health infrastructure, screening for case-finding may result in “positive” cases flooding a fragile, under-resourced health care system [11, 34]. In this study, for example, the majority of women screened (87%) were experiencing high levels of distress. Of course, it is possible that the tool was too sensitive and that it is not appropriate for use in this context. Another consideration is that screening tools which have been developed according to psychiatric diagnostic categories are limited (are not valid or reliable) in fully describing the complexity and multi-dimensional presentation of mental disorders [35].

One strategy would be to increase the cut-point for referral, a large proportion of women who are symptomatic/ moderately depressed would then be excluded. And leaving symptomatic women without care or treatment would ultimately be counterproductive. One way to address this is to deliver preventing and promotive mental health intervention at community level, applied universally to all perinatal women. The Thinking Healthy Intervention is designed for universal delivery providing women with problem solving skills, input about accessing more social support, and behavioural activation—life skills that are of value across health issues and concerns.

Where there are large numbers of women who are symptomatic, the universal intervention approach allows for the greatest coverage of the population, making optimal use of scarce resources. Having said this only three patients (out of 62 identified to be referred) were successfully referred due to situational barriers. Ensuring follow up after screening is a key health system issue in fragile and low resource settings.

There are multiple challenges surrounding the successful implementation of mental health interventions in post-conflict zones. Public health systems, if operational, are faced with competing health-care priorities often accompanied by severely constrained resources [36]. Scaling up interventions and increasing coverage of the population may require partnership with non-governmental and humanitarian organisations. Where there has been a breakdown of government and institutional infrastructure and services, such entities may provide the only resources for care. The results from this study demonstrate that collaboration between public services and iNGOs may allow for greater accessibility to mental health services for those who need them the most by bringing resources to areas where few to none exist.

Another key aspect that also emerged from this study is that there are multiple barriers for women to access mental health care. These barriers may be structural or cultural or both [27]. When mental health interventions are delivered at health facilities, the most vulnerable women may not be able to access care or attend scheduled appointments. They may be prohibited by family members, have financial constraints, or encounter community-based violence or armed conflict. Involving husbands and family members from the start may mitigate some of these barriers. Women’s movement may be controlled by male figures in their household and they may not have access to mobile phones [28]. Where there are strict religious rules and observations, women may be prohibited from freely accessing health care. Therefore, the design of an intervention in such settings needs to consider these constraints and anticipate flexible strategies to deliver maternal mental health care may be required. Delivering screening and counselling in the community should be considered but this might be difficult to establish and for women to access. Home delivery and/or targeting key women leaders in the community may also be an important avenue to explore remaining mindful of cultural and societal norms. Caution needs to be exercised in this regard however due to lack of privacy and confidentiality in the home and could possibly evoke or exacerbate violence against women.

Community-level engagement with all key stakeholder is essential. Increasing community and household awareness around maternal mental health could remove some of the barriers faced by women when seeking help, as well as assist in preventing mental health difficulties from arising. In Afghanistan, for example, it was suggested that initial sensitization sessions could be established at the community level through community “health shuras”, and family health action groups. Information provided to these community level structures could then be disseminated throughout community groups. Alternative ways of reaching women and families could also be considered, for example through radio and social media.

This study demonstrates that THP is a feasible intervention for women in Afghanistan:

- While this study was only a pilot study it appears to show promise in this context with women who participating showing a marked reduction in depressive symptoms;
- The THP appears to be adaptable—the number of sessions was reduced to six and could be integrated into post-partum and infant nutrition programmes or other public health approaches in order to reach more women;
- A core component of the THP is community and household level engagement which may help to address some of the major barriers to women accessing mental health care.

## Limitations

There are limitations of this study that need to be acknowledged. Firstly, the use of self-report measures to collect psychosocial and demographic data may have produced data that could not be verified or that was subject to recall-bias. Secondly, the high drop-out rate of women who participated in the study greatly reduced the sample size for the intervention. Also, as the study was facility-based it is difficult to generalise the findings to other settings in Afghanistan.

## Recommendations

A number of recommendations are clear from this research:

1. While the results of this study are promising, further research is needed to establish the extent to which the THP could be offered universally at community level in Afghanistan. This would raise awareness and normalize mental health in the community, hopefully reduce stigma and increase receptiveness to mental health care;
2. There is compelling evidence for the use of the THP by home visitors in Pakistan and other low income countries, but more research is needed in Afghanistan.
3. A universal approach would strengthen potential support for women at all levels. Context needs to be carefully assessed, and based on this, women requiring specific follow-up can be identified and, depending on available context and resources, either participate in one- on-one follow up at the household level, or group sessions at the health facility, or in the case of women that have suicidal risks or severe depression, referred to specialised services;
4. Providing care during the antenatal period may prevent the onset of postpartum mental health problems and have a positive impact on perinatal outcomes, on maternal health and on child health and development. There is overwhelming evidence that postpartum depression is in most cases preceded by antenatal depression and anxiety [29].

## Conclusion

Given the large numbers of women experiencing perinatal depression, there is an urgent need to provide appropriate and feasible treatment protocols. With few resources available for mental health care, the focus of intervention must shift from a predominant facility-based, clinical service provision to a broader approach. Community-level programmes that include prevention activities, campaigns and home visits according to the context, and that are integrated into existing infant and child health programmes may be a first step in the successful implementation of scalable mental health interventions.

## Data Availability

Data are available upon request.

## References

[1] World Health Organization (2018). Mental Health Atlas 2017.

[2] Jansen S, White R, Hogwood J, Jansen A, Gishoma D, Mukamana D (2015). The "treatment gap" in global mental health reconsidered: sociotherapy for collective trauma in Rwanda. Eur J Psychotraumatol.

[3] Charlson F, van Ommeren M, Flaxman A, Cornett J, Whiteford H, Saxena S (2019). New WHO prevalence estimates of mental disorders in conflict settings: a systematic review and meta-analysis. Lancet.

[4] Lund C, Brooke-Sumner C, Baingana F, Baron EC, Breuer E, Chandra P (2018). Social determinants of mental disorders and the Sustainable Development Goals: a systematic review of reviews. Lancet Psychiatry.

[5] Azale T, Fekadu A, Hanlon C (2018). Postpartum depressive symptoms in the context of high social adversity and reproductive health threats: a population-based study. Int J Ment Health Syst.

[6] Heyningen TV, Myer L, Onah M, Tomlinson M, Field S, Honikman S (2016). Antenatal depression and adversity in urban South Africa. J Affect Disord.

[7] Fisher J, Cabral de Mello M, Patel V, Rahman A, Tran T, Holton S (2012). Prevalence and determinants of common perinatal mental disorders in women in low- and lower-middle-income countries: a systematic review. Bull World Health Organ..

[8] Mitchell AJ, Coyne JC (2007). Do ultra-short screening instruments accurately detect depression in primary care? A pooled analysis and meta-analysis of 22 studies. Br J General Pract.

[9] Ali GC, Ryan G, De Silva MJ (2016). Validated screening tools for common mental disorders in low and middle income countries: a systematic review. PLoS ONE.

[10] United Nations Development Programme (2015). Human development report 2015: work for human development.

[11] Kagee A, Tsai AC, Lund C, Tomlinson M (2013). Screening for common mental disorders in low resource settings: reasons for caution and a way forward. Int Health.

[12] Dixon S, Dantas JA (2017). Best practice for community-based management of postnatal depression in developing countries: A systematic review. Health Care Women Int.

[13] Sikander S, Ahmad I, Atif N, Zaidi A, Vanobberghen F, Weiss HA (2019). Delivering the Thinking Healthy Programme for perinatal depression through volunteer peers: a cluster randomised controlled trial in Pakistan. Lancet Psychiatry.

[14] Sikander S, Lazarus A, Bangash O, Fuhr DC, Weobong B, Krishna RN (2015). The effectiveness and cost-effectiveness of the peer-delivered Thinking Healthy Programme for perinatal depression in Pakistan and India: the SHARE study protocol for randomised controlled trials. Trials.

[15] Loewenberg S (2009). Afghanistan's hidden health issue. The Lancet.

[16] Hunger and Nutrition Commitment Index. Key data for Afghanistan.

[17] Belay T (2010). Building on Early Gains in Afghanistan's Health, nutrition and population sector: challenges and options.

[18] Monitoring and Evaluation Department MoPH, Government of the Islamic Republic of Afghanistan. Afghanistan health indicators fact sheet—August 2008. 2008.

[19] WHO Ua. Skilled birth delivery (SBA)-Joint UNICEF/WHO database 2018.

[20] Health Management Information System (HMIS) Database. Kabul: Ministry of Public Health, Islamic Republic of Afghanistan; 2011.

[21] Vos T, Flaxman AD, Naghavi M, Lozano R, Michaud C, Ezzati M (2012). Years lived with disability (YLDs) for 1160 sequelae of 289 diseases and injuries 1990–2010: a systematic analysis for the Global Burden of Disease Study 2010. Lancet.

[22] Whiteford HA, Degenhardt L, Rehm J, Baxter AJ, Ferrari AJ, Erskine HE (2013). Global burden of disease attributable to mental and substance use disorders: findings from the Global Burden of Disease Study 2010. Lancet.

[23] Kessler RC (2003). Epidemiology of women and depression. J Affect Disord.

[24] WHO. World mental health atlas: Afghanistan. 2014.

[25] Scholte WF, Olff M, Ventevogel P, de Vries GJ, Jansveld E, Cardozo BL (2004). Mental health symptoms following war and repression in eastern Afghanistan. JAMA.

[26] Panter-Brick C, Eggerman M, Gonzalez V, Safdar S (2009). Violence, suffering, and mental health in Afghanistan: a school-based survey. Lancet.

[27] Bizouerne C. Insuffisance en lait maternel et souffrances psychologiques en Afghanistan—Approche psychologique Clinique en situation humanitaire. "Mother Milk Insufficiency and Psychological suffering in Afghanistan—Clinical approach in a humanitarian context. France: Université Bordeaux; 2008.

[28] GSMA. The Mobile Gender Gap: Report 2020. London: GSMA; 2020.

[29] Heron J, O'Connor TG, Evans J, Golding J, Glover V, Team AS (2004). The course of anxiety and depression through pregnancy and the postpartum in a community sample. J Affect Disord.

[30] Meltzer-Brody S, Brandon A (2015). It is time to focus on maternal mental health: optimising maternal and child health outcomes. BJOG.

[31] Parsons CE, Young KS, Rochat TJ, Kringelbach ML, Stein A (2012). Postnatal depression and its effects on child development: a review of evidence from low- and middle-income countries. Br Med Bull.

[32] Rahman A, Patel V, Maselko J, Kirkwood B (2008). The neglected 'm' in MCH programmes–why mental health of mothers is important for child nutrition. Trop Med Int Health.

[33] Honikman S, van Heyningen T, Field S, Baron E, Tomlinson M (2012). Stepped care for maternal mental health: a case study of the perinatal mental health project in South Africa. PLoS Med.

[34] Larvin H, Peckham E, Prady SL (2019). Case-finding for common mental disorders in primary care using routinely collected data: a systematic review. Soc Psychiatry Psychiatr Epidemiol.

[35] Hengartner MP, Lehmann SN (2017). Why psychiatric research must abandon traditional diagnostic classification and adopt a fully dimensional scope: two solutions to a persistent problem. Front Psychiatry.

[36] Fujita N, Zwi AB, Nagai M, Akashi H (2011). A comprehensive framework for human resources for health system development in fragile and post-conflict states. PLoS Med.

