# Correction to: Identifying and treating maternal mental health difficulties in Afghanistan: A feasibility study

**DOI:** 10.1186/s13033-020-00418-y

**Published:** 2020-11-19

**Authors:** Mark Tomlinson, Deepika Chaudhery, Habibullah Ahmadzai, Sofía Rodríguez Gómez, Cécile Bizouerne, Thandi van Heyningen, Mickey Chopra

**Affiliations:** 1 Department of Global Health, Institute for Life Course Health Research, Stellenbosch University, Cape Town, South Africa; 2 School of Nursing and Midwifery, Queens University, Belfast, UK; 3 World Bank, Health, Nutrition and Population Global Practice, South Asia Region, Kabul, Afghanistan; 4 Mental Health and Care Practices, Gender and Protection Department, Action Against Hunger, Kabul, Afghanistan; 5 Division of Epidemiology and Biostatistics, School of Public Health and Family Medicine, University of Cape Town, Cape Town, South Africa; 6 World Bank, Washington, USA

## Correction to: Int J Ment Health Syst (2020) 14:75 10.1186/s13033-020-00407-1

In the original version of the article [1], the surname of the fifth author name was published incorrectly. The corrected author name is given in this correction article. The original article has been corrected.
